# Ternary PM6:Y6 Solar Cells with Single‐Walled Carbon Nanotubes

**DOI:** 10.1002/smsc.202200079

**Published:** 2022-12-25

**Authors:** Laura Wieland, Han Li, Xuning Zhang, Jianhui Chen, Benjamin S. Flavel

**Affiliations:** ^1^ Institute of Nanotechnology Karlsruhe Institute of Technology Hermann-von-Helmholtz-Platz 1 76344 Eggenstein-Leopoldshafen Germany; ^2^ Institute of Materials Science Technische Universität Darmstadt Alarich-Weiss-Straße 2 Darmstadt 64287 Germany; ^3^ Key Laboratory of Optic-Electronic Information and Materials of Hebei Province College of Physics Science and Technology Hebei University Baoding 071002 China

**Keywords:** bulk heterojunctions, carbon nanotubes, morphology, organic solar cells, ternary devices

## Abstract

An organic solar cell with single‐walled carbon nanotubes (SWCNTs) in the photoactive layer is typically a type II heterojunction with the semiconducting SWCNTs acting as the electron donor and C_60_ or other fullerene derivatives as the acceptor. Herein, the performance of solar cells consisting of (6,5) SWCNTs combined with C_60_ and three nonfullerene acceptors is evaluated in a bilayer architecture. SWCNTs are then combined with the donor/acceptor PM6:Y6 in a ternary mixture and both bulk heterojunction and bilayer devices are fabricated. The SWCNTs are found to extend the light absorption of PM6:Y6 solar cells into the infrared but their use must strike a balance between the SWCNT concentration to enhance light absorption and solvent‐induced changes to the morphology of the active layer.

## Graphical abstract

Single‐walled carbon nanotube (SWCNT) solar cells consisting of polymer‐wrapped (6,5) and nonfullerene acceptors such as ITIC‐2F or Y6 are demonstrated in a bilayer architecture. A ternary bulk heterojunction is then developed, which consists of SWCNTs in PM6:Y6 and this leads to an extension of the spectral response into the infrared.

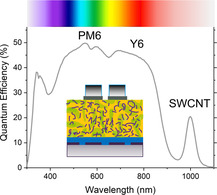

## 1 Introduction

Organic photovoltaic (OPV) research is the fine balance between optimization of the bandgap of new materials, their morphology, and their spectral response to maximize efficiency.^[^ ^1^, ^2^, ^3^, ^4^ ^]^ Semiconducting single‐walled carbon nanotubes (s‐SWCNTs) have tunable bandgaps and a strong optical absorption in the infrared (IR),^[^ ^5^, ^6^ ^]^ and these make them an attractive material for PV, For example: as electrode or hole collection material in silicon and perovskite solar cells,^[^ ^7^, ^8^, ^9^, ^10^, ^11^ ^]^ a counter electrode in dye‐sensitized solar cells^[^ ^12^, ^13^ ^]^ or as a photoactive layer in silicon^[^ ^14^ ^]^ or organic solar cells. Various type II heterojunctions in OPV have been shown to enable exciton dissociation and an electron transfer from the SWCNT donor to acceptor.^[^ ^15^, ^16^, ^17^, ^18^, ^19^, ^20^, ^21^, ^22^, ^23^, ^24^, ^25^ ^]^ Typical s‐SWCNT PV devices have a bilayer architecture, use small diameter carbon nanotubes such as (6,5) or (7,5), and have fullerene C_60_ as the acceptor.^[^ ^19^, ^20^, ^25^, ^26^ ^]^ Using shear force‐mixed (6,5) SWCNTs, Shea et al.^[^ ^20^ ^]^ have achieved an external quantum efficiency (EQE) of 49% in the IR at the characteristic S_11_ transition. However, these bilayer structures suffer from the short exciton diffusion length within carbon nanotubes that limit the film thickness to 5–10 nm^[^ ^16^, ^27^, ^28^ ^]^ and which in turn constrain the maximum achievable light absorption of the devices.

Fullerene derivatives like phenyl‐C_71_‐butyric acid methyl ester (PC_71_BM) have been used to increase the absorption of SWCNT solar cells into the visible and a PCE of 3.2% for a polychiral sample^[^ ^29^ ^]^ and 2.9% for nearly monochiral (6,5) SWCNTs have been achieved.^[^ ^21^ ^]^ In addition, the increased solubility of these fullerene derivatives has facilitated blend mixtures and the formation of bulk heterojunction (BHJ) devices.^[^ ^17^ ^]^ These overcome the SWCNT film thickness limitation but suffer from problems associated with an inhomogeneous and phase‐separated donor/acceptor layer. Likewise, the low photochemical stability^[^ ^30^, ^31^, ^32^ ^]^ and an absorption window of up to 700 nm (PC_71_BM)^[^ ^33^ ^]^ limit the maximum PCE that these BHJs can achieve. The best S_11_ EQE for a SWCNT/PCBM BHJ is 19% and the device itself had a PCE of 1.7%.^[^ ^22^ ^]^ Nonfullerene acceptors (NFAs) are a possible solution because these have a broad spectral absorption, higher extinction coefficient, and tunable energy levels for optimized light capture.^[^ ^1^, ^34^, ^35^ ^]^

Indeed the best performing OPV cells employ NFAs and combinations such as D18:Y6,^[^ ^36^ ^]^ PM6:L8‐BO,^[^ ^4^ ^]^ or PM6:BO‐4Cl^[^ ^37^ ^]^ have been reported to reach PCEs of up to 18.2%. The trade‐off between short circuit current (*J*_SC_) density and open circuit voltage (*V*_OC_) in organic solar cells has been optimized with the use of a ternary device concept. A third material is chosen to broaden the spectral response, regulate the BHJ morphology, improve the *V*_OC_, and reduce the charge recombination in OSCs.^[^ ^38^ ^]^ Here, another acceptor with a lowest unoccupied molecular orbital (LUMO) between the host donor and acceptor is ideal due to a cascade for the electron transfer across the materials such as BTP‐CC^[^ ^39^ ^]^ or PC_71_BM^[^ ^40^ ^]^ in PM6:Y6. Pushing the boundaries of this concept, a PM6:PM7:Y6:PC_71_BM device with double cascading charge transport has achieved 18% efficiency.^[^ ^41^ ^]^ Other strategies to tune performance are preaggregation^[^ ^42^, ^43^ ^]^ or additives to realize a vertical distribution like 1‐chloronaphthalene in D18‐Cl:N3:PC_61_BM.^[^ ^44^ ^]^ Nevertheless, active layers in organic solar cells absorb only up to 1000 nm and have relatively high nonradiative voltage losses compared to other types of solar cells.

To date, only Wang et al.^[^ ^45^ ^]^ have considered the possibility of s‐SWCNT/NFA devices and investigated the exciton dissociation and electron transfer of perylene diimides and ITIC‐2F by internal QE measurements. New NFAs with deeper lying LUMO levels were suggested for future work. However, the working principle of organic solar cells with NFAs is not completely understood and stability in air must be improved. In our work, three widely used nonfullerene acceptors (Y6, ITIC‐2F, and PTCDI‐C8) are combined with polymer‐wrapped small diameter (6,5) SWCNTs. While the EQE intensity at the S_11_ optical bandgap is similar to the reference C_60_ devices, the power conversion efficiency is higher than in s‐SWCNT/fullerene bilayer solar cells. Inspired by the impressive efficiencies of polymer solar cells over 18%, a ternary device is then realized, which consists of PM6/Y6 with additional (6,5) SWCNTs to extend the absorption range into the IR. Both bilayer and bulk heterojunction devices are fabricated and show the advantageous IR extension, while the challenges of performance loss and morphology are discussed.

## 2 Results and Discussion

**Figure 1:**
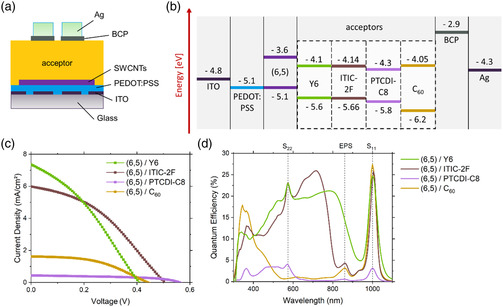
a) Schematic of the solar cell architecture employed and b) an energy level diagram for each of the four acceptors used. c) Current density–voltage (*J*–*V*) and d) external quantum efficiency (EQE) measurements of the assembled solar cells.

**Figure** **1**a shows a schematic of the layer stack used in the solar cells reported by this work. Each consisted of a patterned indium tin oxide (ITO) glass substrate to which a layer of poly(3,4‐ethylenedioxythiophene):polystyrene sulfonate (PEDOT:PSS) and a layer of (6,5) SWCNTs were sequentially spin coated. This was followed by the deposition of one of the four different acceptor layers and evaporation of the electron transport layer bathocuproine (BCP) and a silver electrode to complete the device. The four different acceptors were C_60_, which were thermally evaporated, and the nonfullerene acceptors (NFAs): Y6, ITIC‐2 F, and PTCDI‐C8, which were all spin cast from a chlorobenzene solution. The chemical structure of each of the NFAs and an absorption spectrum of the (6,5) SWCNTs in toluene is shown in Figure S1 and S2, Supporting Information. The (6,5) SWCNTs were separated from the CoMoCAT raw material using the selective polymer PFO‐BPy^[^ ^46^, ^47^ ^]^ and shear force mixing was used to maximize their length and minimize the defect density. This ensures fewer exciton quenching sites on the nanotubes and, thus, longer lifetimes and higher quantum yields.^[^ ^48^, ^49^, ^50^, ^51^ ^]^ In all cases, it was important to have a low PFO‐BPy polymer content to ensure good contact between SWCNTs and the acceptor.^[^ ^52^, ^53^ ^]^ A reduction in the polymer content was achieved by filtration and rinsing the (6,5) SWCNTs with toluene. Absorption spectra of the original and concentrated sample are shown in Figure S2, Supporting Information. SWCNT films were prepared by multiple spin coating steps from a concentrated solution in toluene to achieve thicknesses of 8–18 nm.

As shown in Figure 1b, each of the acceptors formed a type II heterojunction with the (6,5) SWCNTs and in a simplified model, the energetic offset between the LUMO of the donor and that of the acceptor can be used to predict the occurrence of exciton dissociation at the interface.^[^ ^54^ ^]^ To be precise, the minimum energy that is required for exciton dissociation at the heterojunction is the thermodynamic driving force (Δ*G*), which can be calculated by Δ*G* = |IP_D_ − EA_A_| − [*E*_el_ − *E*_b_].^[^ ^15^, ^55^ ^]^ The absolute value of the difference between the ionization potential of the donor (IP_D_) and the electron affinity of the acceptor (EA_A_) minus the exciton energy. This includes the electronic bandgap of the SWCNT donor (*E*_el_) and the exciton binding energy (*E*_b_). Solar energy conversion only occurs if Δ*G* < 0. The LUMO position of the (6,5) SWCNT, as shown in Figure 1b, was calculated based on the optical bandgap and *E*_b_;^[^ ^54^ ^]^ therefore, the required minimum energy is denoted as LUMO offset. Exciton binding energies within SWCNTs have been estimated to range from 0.2 to 0.5 eV^[^ ^56^, ^57^ ^]^ and a reorganization energy of 130 meV has been shown to be required.^[^ ^58^ ^]^ Current density–voltage (*J*–*V*) curves and performance values for each (6,5)/acceptor combination are shown in Figure 1c and **Table** **1**, respectively. Overall, the two solar cells containing ITIC‐2 F and Y6 performed the best and these had efficiency values of 1.16% and 1.09%, respectively. Despite an efficiency of ≈1% being low for the broad organic solar cell field, it is important to remember that the maximum efficiencies reported in the field of SWCNT solar cells are ≈3%.^[^ ^21^, ^29^ ^]^ It is also a significant improvement compared to the use of C_60_, which had an efficiency of 0.33%. Shea and Arnold reported a PCE of 1.02% for their (6,5)/C_60_ device.^[^ ^19^ ^]^ The fill factor (FF) and *V*_OC_ of all (6,5)/acceptor combinations were 34–51% and 0.41–0.56 V, but the better performance for these two NFAs can mostly be attributed to the improved *J*_SC_, which was almost four times higher than for C_60_.

**Table 1:** Solar cell performance parameters for the ITO/PEDOT:PSS/(6,5)/acceptor/BCP/Ag devices

Layer stack [ITO/PEDOT:PSS/(6,5)/…/BCP/Ag]	*J*_SC_ [mA cm^−^^2^]	*V*_OC_ [mV]	FF [%]	Efficiency [%]	Peak EQE at S_11_ of (6,5) [%]
C_60_	1.62	441.6	46.03	0.33	27.39
Y6	7.35	408.2	33.5	1.01	24.67
ITIC‐2 F	5.97	502.9	38.61	1.16	25.75
PTCDI‐C8	0.44	560.8	51.02	0.13	3.15

Figure 1d shows an EQE measurement for each of the solar cell devices. The sharp peaks at ≈1000 and ≈575 nm are associated with the first (S_11_) and second (S_22_) optical transitions of (6,5) SWCNTs; the broad peak at ≈860 nm is the exciton–phonon sideband (EPS) for the S_11_ transition and all other major features are associated with the acceptor used. As such, the reason for the improved *J*_SC_ is apparent. For (6,5)/C_60_ solar cells, nearly, the entire visible spectrum is unabsorbed, whereas ITIC‐2F or Y6 is designed to capture most of the visible and near‐IR light. For reference, thin films of ITIC‐2F and Y6 on glass have been measured in transmittance, as shown in Figure S3, Supporting Information, and have an absorption maximum located at 725^[^ ^59^ ^]^ and 810 nm,^[^ ^44^ ^]^ respectively. While ITIC‐2F only absorbs light up to 800 nm, Y6 has an absorption tail reaching nearly 1000 nm. The integrated current of Y6 with 4.34 mA cm^−2^ and ITIC‐2F with 4.31 mA cm^−2^ in the visible region (400–800 nm) illustrates the superiority of the NFAs compared to C_60_ with 0.41 mA cm^−^^2^. A further figure of merit used in the SWCNT solar cell field is the peak EQE at the S_11_ position of the SWCNTs. For the (6,5)/Y6, (6,5)/ITIC‐2F, and (6,5)/C_60_ solar cells, the peak EQE at S_11_ was 24.7%, 25.8%, and 27.4%, respectively, and this is due to their similar LUMO offset of 0.5, 0.54, and 0.45 eV. These EQE results are in agreement with the highly efficient SWCNT solar cells from Classen et al.,^[^ ^21^ ^]^ who achieved 26% with their (6,5)/PC_71_BM devices. In contrast, the (6,5)/PTCDI‐C8 solar cell has an LUMO offset of 0.7 eV, which means that junction is in the Marcus inverted region and the peak EQE is consequently considerably lower at only 3.2%.

**Figure 2:**
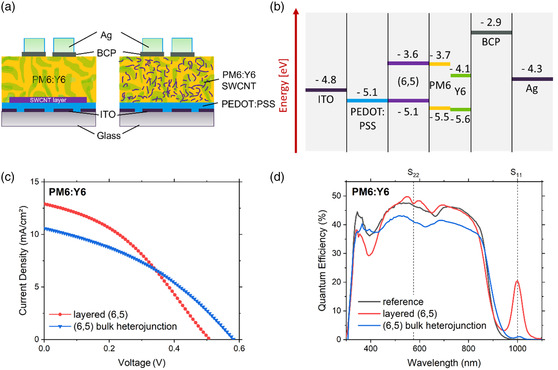
a) Schematic of the layered and bulk heterojunction solar cell architecture employing (6,5) SWCNTs and PM6:Y6. b) The corresponding energy level diagram. c) Current density–voltage (*J*–*V*) and d) external quantum efficiency (EQE) measurements of the assembled solar cells.

Considering Y6 absorbs light over the broadest spectral range (600–1000 nm) of all the acceptors tested and its appropriate LUMO offset to (6,5) SWCNTs for exciton dissociation, we now turn to the development of a ternary device. In these solar cells, we combine Y6 with two donors: PM6, which absorbs light in the spectral range of 500–700 nm (max 610 nm)^[^ ^44^ ^]^ and (6,5) SWCNTs for their IR absorption (Figure S1 and S3, Supporting Information). As shown in **Figure** **2**a, two different solar cell architectures were tested. The first was a layered structure like the devices, as shown in Figure 1a, in which PM6:Y6 was directly spin cast on top of the (6,5) SWCNTs and the second was a BHJ. For the BHJ, 75 μg of (6,5) SWCNTs^[^ ^60^ ^]^ was directly added in a 5:3 ratio to the PM6:Y6 solution in chloroform before spin coating. For the layered structure, the SWCNT film had a thickness of 18 nm. An energy level diagram of these solar cells is shown in Figure 2b, where a cascade of energy levels facilitates charge separation with holes easily moving toward the ITO cathode and electrons to the silver anode. PM6 is also a donor and concurrently serves as facilitator between (6,5) and Y6 with its close LUMO or highest occupied molecular orbital (HOMO) level, respectively (Figure 2b). Excitons generated by CNTs can relax to the lower PM6‐HOMO to enable charge splitting. *J–V* curves and performance values for these two architectures and a PM6:Y6 solar cell without SWCNTs as a control are shown in **Table** **2** and Figure 2c and S4, Supporting Information. The efficiency of the layered device was 2.44% and for the BHJ, it was 2.27%.

**Table 2:** Solar cell performance parameters for layered and bulk heterojunction ITO/PEDOT:PSS/(6,5)/PM6:Y6/BCP/Ag devices

Layer stack [ITO/PEDOT:PSS/…/BCP/Ag]	*J*_SC_ [mA cm^−^^2^]	*V*_OC_ [mV]	FF [%]	Efficiency [%]	Peak EQE at S_11_ of (6,5) [%]
PM6:Y6 (bulk heterojunction)	16.65	816.1	59.43	8.07	–
(6,5) layered	12.86	511.0	37.13	2.44	20.49
(6,5) bulk heterojunction	10.57	581.8	36.92	2.27	1.04

**Figure 3:**
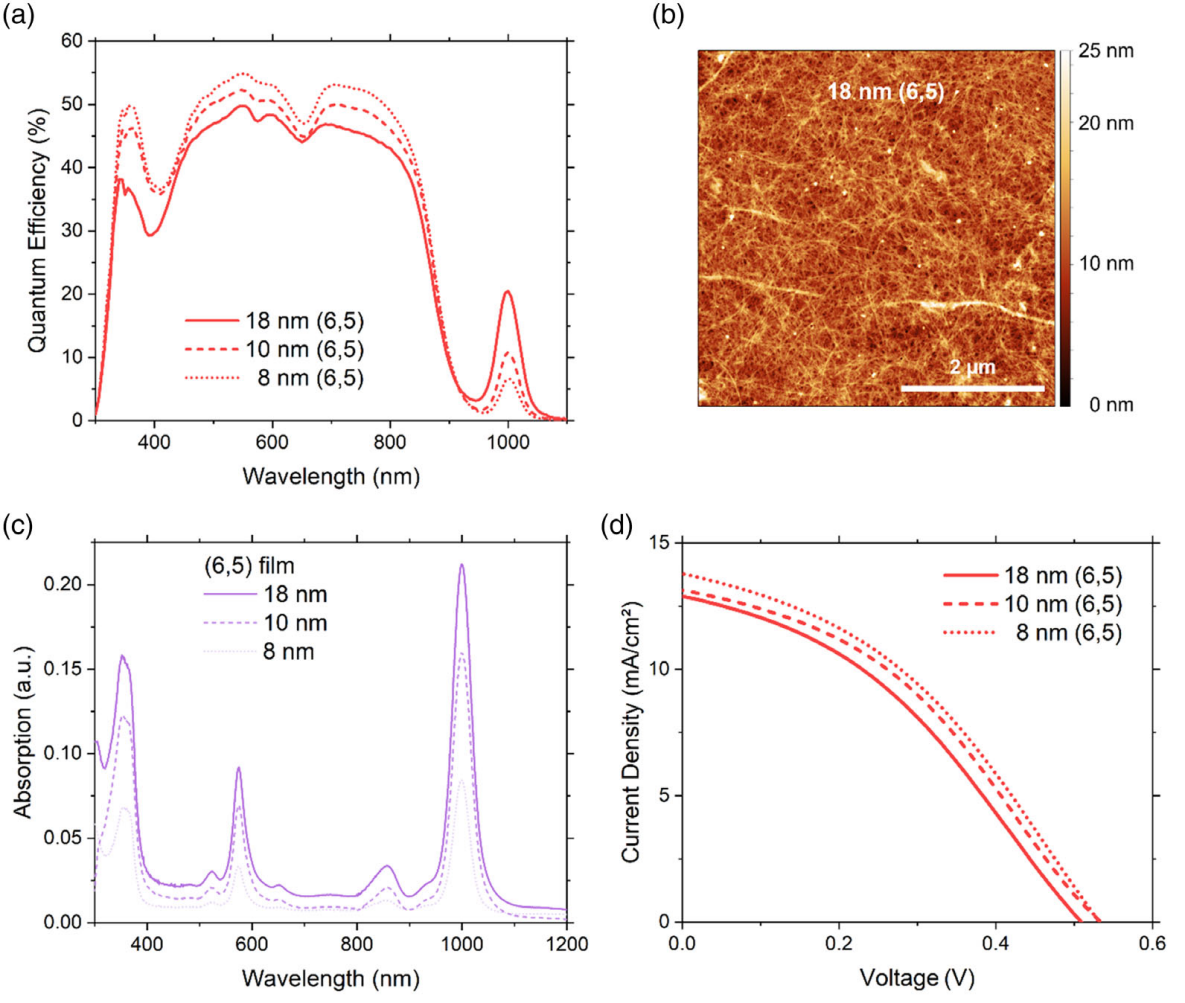
a) EQE measurements of ITO/PEDOT:PSS/(6,5):PM6:Y6/BCP/Ag bilayer devices with increasing thickness of the spin‐cast (6,5) nanotubes. b) AFM image of the interwoven CNT network for an 18 nm thick layer of (6,5) SWCNTs. c) Film absorption measurements for different film thickness of (6,5) SWCNTs and d) the corresponding current density–voltage measurements.

These values compare favorably with best in the field SWCNT solar cells from Gong et al.^[^ ^29^ ^]^ or Classen et al.^[^ ^21^ ^]^ who demonstrated efficiencies of ≈3%. The polychiral device in a BHJ achieved 3.2%, whereas the layered (6,5) device with PC_71_BM from Classen reaches 2.9%. The layered device in this work also had the higher *J*_SC_ of 12.9 mA cm^−^^2^ vs 10.6 mA cm^−^^2^ for the BHJ. EQE measurements reveal the layered device to follow a similar shape to the PM6:Y6 reference cell albeit with an extra peak in the IR at ≈1000 nm from the (6,5) SWCNTs. Here, the peak EQE at S_11_ was 20.5%. By integrating the EQE curve between 900 and 1100 nm, this additional peak from the SWCNTs in the IR equates to 0.65 mA cm^−2^. Relative to the PM6:Y6 reference cell, a dip in the EQE at 575 nm can also be seen for the layered device. This corresponds to S_22_ of (6,5) and is a result of the light first passing through the SWCNT before reaching the PM6:Y6. The excitonic relaxation time from S_22_ to S_11_ is ≈40 fs,^[^ ^61^, ^62^ ^]^ whereas charge transfer from (6,5) CNTs to C_60_ occurs within ≈120 fs.^[^ ^63^ ^]^ Based on the different time scales, the probability of charge transfer from S_22_ to C_60_ is much lower than the relaxation to the S_11_ and then to C_60_. As such, photocurrent generation has been reported for S_22_^[^ ^24^, ^26^, ^64^ ^]^ but only over an intermediate transition to S_11_. Here, S_22_ appears as a dip in the EQE because evidently the loss in current due to a reduction in the light absorbed by PM6:Y6 is not balanced by absorption of the SWCNTs. A positive and negative contribution to EQE from the S_11_ and S_22_ transitions of SWCNTs, respectively, can likewise be seen for the BHJ, albeit the magnitude of these peaks is significantly reduced. This is primarily due to the relative concentration of SWCNTs present in each of the devices. For the layered device, 330 μL of (6,5) SWCNTs with an estimated concentration of 89 μg mL^−1^ (Figure S2, Supporting Information) was used, whereas for the BHJ, 500 μL of SWCNTs was dried and redissolved in the PM6:Y6 solution. Both approaches have their associated losses, but we estimate the number of CNTs per cm^2^ to be higher for the layered than for the BHJ devices. This assumption is supported by the film absorptivity of the (6,5) SWCNTs at S_11_, as shown in **Figure** **3**c, which shows a higher optical density for the layer architecture compared to the ternary (6,5)/PM6/Y6, as shown in Figure S5b, Supporting Information.

It follows that the performance of those solar cells with a layer of (6,5) SWCNTs is influenced by the film thickness. As shown in Figure 3a, an increase from ≈8 to 10 nm and 18 nm of SWCNT layers rises the peak EQE at S_11_ of the SWCNTs from 6.7% to 10.9% to 20.5%. The thickness of these films was determined by atomic force microscopy (AFM), as shown in Figure 3b, and this corresponds to an increase in optical density of 0.013 per nm, as shown in Figure 3c. In all cases, the SWCNT film was a closed packed layer. Unfortunately, despite the increase in EQE at S_11_ for a thicker film of (6,5) SWCNTs, the value of *J*_SC_, and, by extension, the efficiency of the solar cells were reduced with increasing film thickness, as shown in Figure 3d and **Table** **3**.

**Table 3:** Solar cell performance parameters for layered ITO/PEDOT:PSS/(6,5)/PM6:Y6/BCP/Ag devices with varying thickness of the SWCNT layer

(6,5) layer thickness [ITO/PEDOT:PSS/(6,5)/PM6:Y6/BCP/Ag]	*J*_SC_ [mA cm^−^^2^]	*V*_OC_ [mV]	FF [%]	Efficiency [%]	Peak EQE at S_11_ of (6,5) [%]
18 nm	12.86	511.0	37.13	2.44	20.49
10 nm	13.11	539.2	38.16	2.70	10.85
8 nm	13.75	531.4	38.73	2.83	6.70

Like the strong absorption of light from S_22_, which prevents light reaching the PM6:Y6, a thick film of SWCNTs is also associated with an increased scattering background (Figure 3c). This too will reduce the measured *J*_SC_ and it is indeed seen in the EQE measurements, as shown in Figure 3a. An optimum thickness of the SWCNT film is defined by the exciton diffusion length, which has been determined by ultrafast spectroscopy^[^ ^28^, ^65^ ^]^ or photocurrent measurements in bilayer devices.^[^ ^16^, ^27^, ^28^ ^]^ This is seen by an EQE signal and *J*_SC_, which increase linearly up to film thicknesses of 5–15 nm, but then steeply decreases for thicker films.^[^ ^19^, ^20^, ^27^ ^]^ The same behavior was identified by our group for (6,5) films from aqueous solutions with a maximum EQE of 8% for a 18 nm thick SWCNT layer.^[^ ^25^ ^]^ In this work, the highest EQE response of 20.5%, as shown in Figure 3a, was once again achieved again by an 18 nm‐thick film but with (6,5) polymer‐wrapped CNTs. The limited diffusion length is another reason for a reduced EQE signal in the visible region because the separated charge (hole) from the PM6:Y6 interface has to overcome an additional CNT film barrier.

**Figure 4:**
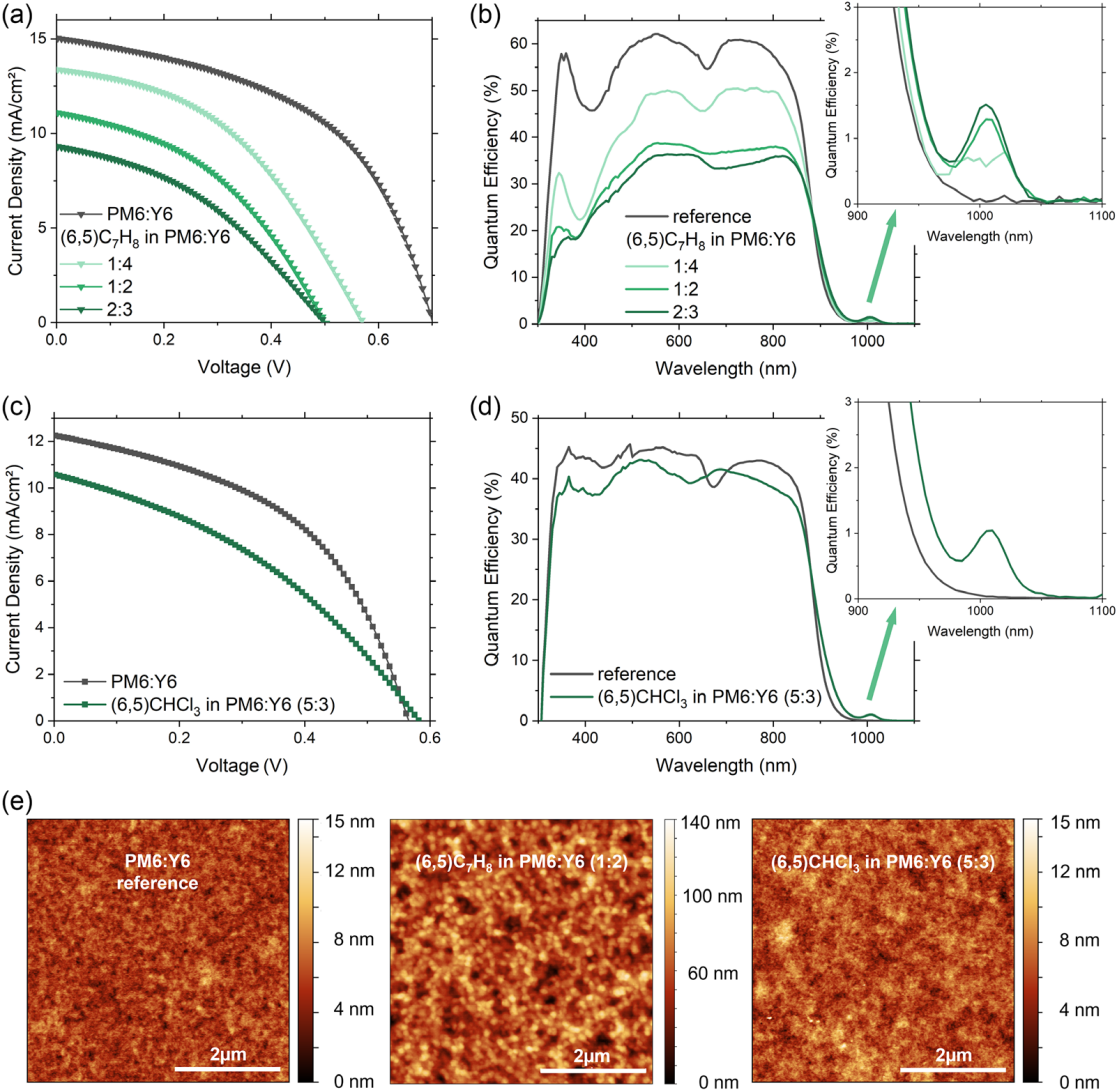
a) Current–voltage measurements of bulk heterojunction (6,5):PM6:Y6 solar cells prepared with a different ratio of PM6:Y6 and with the SWCNTs in toluene (C_7_H_8_). b) The corresponding external quantum efficiency measurements. c) Current–voltage measurements of bulk heterojunction (6,5):PM6:Y6 solar cells prepared with the SWCNTs in chloroform (CHCl_3_) and used to disperse PM6/Y6. d) The corresponding external quantum efficiency measurements. e) AFM images of surface picture of PM6:Y6 prepared in a ratio of 5:6 and with the inclusion of (6,5) SWCNTs in toluene or chloroform.

For a BHJ, issues related to the exciton diffusion length of the SWCNTs and a filtering of the light that eventually reaches the PM6:Y6 are minor concerns. Nevertheless, the amount of (6,5) SWCNTs used was found to have a large impact on the overall device performance. As shown in **Table** **4** and **Figure** **4**a, an increase of (6,5) SWCNTs in the PM6:Y6 bulk layer from a ratio of 1:4–2:3 caused a reduction of *J*_SC_ and *V*_OC_ of up to a third. For example, *J*_SC_ is reduced from 15.0 mA cm^−2^ for the reference PM6:Y6 device to 9.3 mA cm^−2^ for a 2:3 ratio of SWCNTs. These devices were prepared by directly adding the (6,5) SWCNT solution in toluene to the PM6:Y6 in chloroform at the desired volume:volume ratio. This has the effect of diluting the PM6:Y6 solution, which leads to an overall diminishing EQE of the PM6:Y6 layer, as shown in Figure 4b. Dilution of PM6:Y6 with pure toluene shows the same reduction in *J*_SC_ and approves the decreased EQE intensity in the visible region (see Figure S6 and Table S1, Supporting Information). However, the quantum efficiency at S_11_ is increasing with higher (6,5) content in the PM6:Y6 solution, more precisely from 0.80% for the 1:4 ratio up to 1.29% (1:2) and 1.51% (2:3).

**Table 4:** Solar cell performance parameters for bulk heterojunction ITO/PEDOT:PSS/(6,5):PM6:Y6/BCP/Ag devices with varying SWCNT content

Layer stack [ITO/PEDOT:PSS/…/BCP/Ag]	*J*_SC_ [mA cm^−^^2^]	*V*_OC_ [mV]	FF [%]	Efficiency [%]	Peak EQE at S_11_ of (6,5) [%]
PM6:Y6	15.01	700.0	50.25	5.28	–
(6,5)C_7_H_8_ in PM6:Y6 (1:4)	13.36	570.8	43.14	3.29	0.79
(6,5)C_7_H_8_ in PM6:Y6 (1:2)	11.08	500.3	41.63	2.31	1.41
(6,5)C_7_H_8_ in PM6:Y6 (2:3)	9.30	500.2	38.39	1.78	1.52
PM6:Y6	12.24	565.4	47.61	3.2947	–
(6,5)CHCl_3_ in PM6:Y6 (5:3)	10.57	581.8	36.92	2.27	1.04

The addition of toluene was also found to change the morphology of the PM6:Y6 layer. As shown in Figure 4e, the PM6:Y6 became phase separated after addition of the toluene solution. Increasing the ratio of toluene in PM6:Y6 chloroform solution causes the formation of large polymer aggregates and rougher surfaces. This is reflected in the root mean square (RMS) values, which rise from 15.4 nm (1:4) to 17.6 nm (1:2) and 20.7 nm (2:3), which is an order of magnitude higher compared to the PM6:Y6 reference with 1.28 nm (Figure S7, Supporting Information).

In the literature, PM6:Y6 solar cells with an optimized film thickness (250 nm) have an estimated domain size of 44 nm for the Y6 phase.^[^ ^66^ ^]^ The exciton diffusion length of Y6 was determined as 29–37 nm^[^ ^67^, ^68^, ^69^ ^]^ and 47 nm for ITIC‐2F,^[^ ^69^ ^]^ which is in the standard range for NFAs. However, active layer thickness of 100–200 nm is necessary to absorb light efficiently, so BHJ or layer‐by‐layer solar cells are preferred to bilayers.^[^ ^70^, ^71^ ^]^ The complex morphology in these samples can be optimized by solvent additives^[^ ^44^, ^72^, ^73^ ^]^ or thermal annealing. Larger domain sizes can also hinder diffusion processes and reduce *J*_SC_, while ideal morphology is nearly insensitive to variable exciton diffusion length.^[^ ^70^ ^]^ An extraordinarily long diffusion length of 330 nm in PM6:Y6 solar cells was reported by Tokmoldin et al.^[^ ^74^ ^]^ highlighting the enhanced carrier diffusion in thicker‐doped active layers. To optimize the solar cells in this work, the (6,5) SWCNTs were dispersed in chloroform with the aid of sonication, after original solvent toluene had been evaporated. This (6,5) chloroform solution was then the stock solution for the addition of PM6:Y6 powders in their 1:1.2 ratio. This allowed us to maintain a fixed PM6:Y6 concentration and avoid phase separation due to solvent miscibility. As a result, after the integration of SWCNTs, the *V*_OC_ of the device remained comparable to the PM6:Y6 reference and the size of the polymer aggregates was significantly reduced (Figure 4e). However, this is still rougher than for PM6:Y6 alone, and RMS of 1.48 nm is compared to reference 1.28 nm (Figure S6, Supporting Information), which are reflected in a reduction of *J*_SC_ and EQE (Figure 4c,d). The S_11_ quantum efficiency is thereby diminished to 1.0% for the (6,5)CHCl_3_ in PM6:Y6 (5:3) sample. Clearly, also the addition of SWCNTs, changes the domain sizes of PM6:Y6 and will need to be improved in the future.

## 3 Conclusion

NFAs are suitable for SWCNT solar cells considering the minimum energy necessary for exciton dissociation. Among the tested (6,5)/NFA solar cells, ITIC‐2F has the best performance in terms of efficiency, while Y6 has the broadest spectral absorption and, thus, the highest *J*_SC_. In addition to bilayer devices, ternary architectures of (6,5)/PM6/Y6 were investigated with single chirality (6,5) carbon nanotubes as second donor. EQEs over 20% were achieved with an 18 nm (6,5) layer in the following stack ITO/PEDOT:PSS/(6,5)/PM6:Y6/BCP/Ag. The short exciton diffusion length of SWCNTs limits the extra energy harvesting in the IR, therefore BHJs were prepared. However, phase separation was observed by adding polymer‐wrapped (6,5) in toluene directly to the chloroform PM6:Y6 solution. Preparation in a single solvent resolved the morphology and crystallinity issues, but it also made it more difficult to integrate a high concentration of SWCNTs into the system. In summary, all prepared devices showed an absorption extension into the IR, while the layered architecture achieves high EQE response BHJs that suffer from morphology and recombination.

The absorption extension could be in principle broader and reaching wider into the IR if SWCNT mixtures are used instead of single‐chirality SWCNTs. The major problem in a mixed CNT network is the energy transfer from the larger to the smaller bandgap nanotube, which typically results in a dark state that cannot contribute to solar cell performance. Therefore, single chirality small diameter (larger bandgap) nanotubes are prepared by SFM for longer and less defected CNTs.

Nevertheless, BHJs with SWCNTs are a promising architecture for the future. The bottleneck will now only be the amount of SWCNTs that can be integrated into the PM6:Y6 without causing a phase separation or any dramatic change to the morphology. In the future, this could possibly be achieved by making modifications to the side chains of PM6 or Y6 to make them more compatible with SWCNTs. Ideally, NFAs or the polymer donors will be designed in such a way that SWCNTs can be directly sorted or dispersed by a PM6 or Y6 derivative.

## 4 Experimental Section

4.1

4.1.1

##### SWCNT Solution

A polymer‐wrapped (6,5) suspension was prepared by shear force mixing^[^ ^48^ ^]^ of 40 mg of CoMoCAT (Sigma‐Aldrich, 773 735 lot no. MKBZ1159V) with 55 mg poly[(9,9‐dioctylfluorenyl‐2,7diyl)‐alt*‐co*‐(6,6’‐(2‐20‐bipyridine))] (PFO‐BPy) (American Dye Source, lot no. 19L014A1) in 110 mL toluene (99.5%, ACS reagent, Sigma‐Aldrich) for 65 h. Subsequently, the suspension was centrifuged in a SW‐40‐Ti rotor (Beckman‐Coulter Optima L‐80 XP) at 45 560 g for 30 min at 20 °C. The supernatant was concentrated by filtration (nylon membrane, 0.2 mm pore size), rinsed with toluene, and redispersed to reduce the excess polymer content (Figure S2, Supporting Information).

##### Device Fabrication

Prestructured ITO substrates (Psiotec, 15 Ω sq^−1^) were cleaned with acetone and detergent, followed by water and isopropanol. After drying, PEDOT:PSS (AI 4083, Ossila) was filtered (Millex‐HV, 0.45 μm, Merck) and spin cast on the ITO with 3,000 rpm for 30 s, followed by the annealing at 150 °C for 15 min in a glove box. For the layered devices, polymer‐wrapped (6,5) SWCNT were spin cast directly on top of the PEDOT:PSS (55 μL, 600 rpm for 20 s followed by 1500 rpm for 5 s) and annealed at 110 °C for 7 min. The next layer was the acceptor, which in the case of the C_60_ fullerene 100 nm was evaporated (99.9+%; Sigma‐Aldrich, lot MKCK0541) or a 40 nm layer of *N,N*′‐dioctyl‐3,4,9,10‐perylene dicarboximide (PTCDI‐C8, Sigma‐Aldrich, lot MKBV5102V). While the other NFAs are spin cast from chlorobenzene solutions: 3,9‐bis(2‐methylene‐((3‐(1,1‐dicyanomethylene)‐6,7‐difluoro)‐indanone))‐5,5,11,11‐tetrakis(4‐hexylphenyl)‐dithieno[2,3‐d:2′,3′‐d′]‐s‐indaceno[1,2‐b:5,6‐b′]dithiophene) (ITIC‐2 F, 10 mg mL^−1^, 1000 rpm 20 s, Ossila, lot M2075A1) and 2,2'‐((2Z,2'Z)‐((12,13‐bis(2‐ethylhexyl)‐3,9‐diundecyl‐12,13‐dihydro‐[1,2,5]thiadiazolo[3,4‐e]thieno[2″,3″:4′,5′]thieno[2′,3′:4,5]pyrrolo[3,2‐g]thieno[2′,3′:4,5]thieno[3,2‐b]indole‐2,10‐diyl)bis(methanylylidene))bis(5,6‐difluoro‐3‐oxo‐2,3‐dihydro‐1H‐indene‐2,1‐diylidene))dimalononitrile (Y6, 12 mg mL^−1^, 3,000 rpm 30 s, Ossila, lot M2200A1).

Instead of an acceptor, the layer on top of the (6,5) thin film consisted of the polymer poly[(2,6‐(4,8‐bis(5‐(2‐ethylhexyl‐3‐fluoro)thiophen‐2‐yl)‐benzo[1,2‐b:4,5‐b′]dithiophene))‐*alt*‐(5,5‐(1′,3′‐di‐2‐thienyl‐5′,7′‐bis(2‐ethylhexyl)benzo[1′,2′‐c:4′,5′‐c′]dithiophene‐4,8‐dione))] (PM6, Ossila, lot M2150A8) and Y6 mixed in chloroform witch an total concentration of 16 mg mL^−1^ (1:1.2).

The BHJ device consisted of PM6:Y6 (1:1.2) in chloroform, which was spin cast on top of PEDOT:PSS. For the (6,5)C_7_H_8_ sample, the polymer‐wrapped (6,5) in toluene is added into PM6:Y6 in the declared ratios (1:4, 1:2, and 2:3). To avoid mixing two solvents, 500 μL (6,5) in toluene was first heated up to evaporate and redisperse the CNTs in chloroform. Again, PM6:Y6 (1:1.2) was added into the solution before spin casting it on the PEDOT:PSS. To complete device fabrication, 10 nm of BCP (>99.5%, Ossila) was thermally evaporated followed by the 100 nm thick silver electrode. The active area of a cell was 0.105 cm^2^ and all devices were characterized under ambient conditions.

##### Characterization

The *J*–*V* characteristics were measured with a Keithley 2601B source meter under AM1.5 G illumination from a LOT‐QuantumDesign solar simulator (450–1000 W Xe Arc Lamp). EQE measurements were conducted using the integrated system SpeQuest Quantum Efficiency from Rera solutions. Calibration was performed with Si 250–100 nm and Ge 700–1800 nm diodes. UV–Vis‐near infrared (NIR) absorbance spectra of the polymer‐wrapped SWCNT solutions were collected on a Cary 500 spectrometer from 1400 to 200 nm in a 1 mm quartz cuvette. Films for absorption measurements were prepared on glass microscope slides following the same spin coating procedures mentioned above.

##### Atomic Force Microscopy

Topographies were recorded with a Dimension Icon, Bruker with NSC 19 cantilevers (μmasch) with a resonance frequency of 65 kHz and a force constant of 0.5 N m^−1^. Imaging was performed in the repulsive regime with standard tapping mode in air and a resolution of 1024 lines. All topographies were evaluated using open‐source Gwyddion, for example, the determination of the RMS.

## Conflict of Interest

The authors declare no conflict of interest.

## Supporting information

Supplementary Material

## Data Availability

The data that support the findings of this study are available from the corresponding author upon reasonable request.

## References

[1] D. Luo , W. Jang , D. D. Babu , M. S. Kim , D. H. Wang , A. K. K. Kyaw , J. Mater. Chem. A 2022, 10, 3255.

[2] Y. Wang , J. Lee , X. Hou , C. Labanti , J. Yan , E. Mazzolini , A. Parhar , J. Nelson , J.-S. Kim , Z. Li , Adv. Energy Mater. 2021, 11, 2003002.

[3] G. Han , Y. Yi , Acc. Chem. Res. 2022, 55, 869. 35230078 10.1021/acs.accounts.1c00742

[4] X. N. Zhang , C. Li , J. Q. Xu , R. Wang , J. L. Song , H. Zhang , Y. X. Li , Y. N. Jing , S. L. Li , G. B. Wu , J. Zhou , X. Li , Y. Y. Zhang , X. Li , J. Q. Zhang , C. F. Zhang , H. Q. Zhou , Y. M. Sun , Y. Zhang , Joule 2022, 6, 444.

[5] A. Jorio , G. Dresselhaus , M. S. Dresselhaus , Carbon Nanotubes, Springer, Berlin Heidelberg, Germany 2008.

[6] H. Kataura , Y. Kumazawa , Y. Maniwa , I. Umezu , S. Suzuki , Y. Ohtsuka , Y. Achiba , Synth. Met. 1999, 103, 2555.

[7] J. Chen , D. D. Tune , K. Ge , H. Li , B. S. Flavel , Adv. Funct. Mater. 2020, 30, 2000484.

[8] J. H. Chen , L. Wan , H. Li , J. Yan , J. K. Ma , B. Sun , F. Li , B. S. Flavel , Adv. Funct. Mater. 2020, 30, 2004476.

[9] R. Ihly , A.-M. Dowgiallo , M. Yang , P. Schulz , N. J. Stanton , O. G. Reid , A. J. Ferguson , K. Zhu , J. J. Berry , J. L. Blackburn , Energy Environ. Sci. 2016, 9, 1439.

[10] L. Fagiolari , F. Bella , Energy Environ. Sci. 2019, 12, 3437.

[11] S. N. Habisreutinger , T. Leijtens , G. E. Eperon , S. D. Stranks , R. J. Nicholas , H. J. Snaith , J. Phys. Chem. Lett. 2014, 5, 4207. 26278955 10.1021/jz5021795

[12] T. Chen , L. Qiu , Z. Cai , F. Gong , Z. Yang , Z. Wang , H. Peng , Nano Lett. 2012, 12, 2568. 22500591 10.1021/nl300799d

[13] Z. Yang , M. Liu , C. Zhang , W. W. Tjiu , T. Liu , H. Peng , Angew. Chem. Int. Ed. 2013, 52, 3996. 10.1002/anie.201209736 23401014

[14] D. D. Tune , N. Mallik , H. Fornasier , B. S. Flavel , Adv. Energy Mater. 2019, 10, 1903261.

[15] J. L. Blackburn , ACS Energy Lett. 2017, 2, 1598.

[16] D. J. Bindl , M. Y. Wu , F. C. Prehn , M. S. Arnold , Nano Lett. 2011, 11, 455. 21166422 10.1021/nl1031343

[17] D. J. Bindl , A. S. Brewer , M. S. Arnold , Nano Res. 2011, 4, 1174.

[18] G. I. Koleilat , M. Vosgueritchian , T. Lei , Y. Zhou , D. W. Lin , F. Lissel , P. Lin , J. W. To , T. Xie , K. England , Y. Zhang , Z. Bao , ACS Nano 2016, 10, 11258. 28024326 10.1021/acsnano.6b06358

[19] M. J. Shea , M. S. Arnold , Appl. Phys. Lett. 2013, 102, 243101.

[20] M. J. Shea , J. L. Wang , J. T. Flach , M. T. Zanni , M. S. Arnold , APL Mater. 2018, 6, 056104.

[21] A. Classen , L. Einsiedler , T. Heumueller , A. Graf , M. Brohmann , F. Berger , S. Kahmann , M. Richter , G. J. Matt , K. Forberich , J. Zaumseil , C. J. Brabec , Adv. Energy Mater. 2018, 8, 1801913.

[22] Y. Ye , D. J. Bindl , R. M. Jacobberger , M. Y. Wu , S. S. Roy , M. S. Arnold , Small 2014, 10, 3299. 24719253 10.1002/smll.201400696

[23] R. M. Jain , R. Howden , K. Tvrdy , S. Shimizu , A. J. Hilmer , T. P. McNicholas , K. K. Gleason , M. S. Strano , Adv. Mater. 2012, 24, 4436. 22740144 10.1002/adma.201202088

[24] M. Pfohl , K. Glaser , J. Ludwig , D. D. Tune , S. Dehm , C. Kayser , A. Colsmann , R. Krupke , B. S. Flavel , Adv. Energy Mater. 2016, 6, 1501345.

[25] L. Wieland , C. Rust , H. Li , M. Jakoby , I. Howard , F. Li , J. Shi , J. Chen , B. S. Flavel , Carbon 2021, 184, 828.

[26] D. J. Bindl , M. S. Arnold , J. Phys. Chem. C 2013, 117, 2390.

[27] D. J. Bindl , M. J. Shea , M. S. Arnold , Chem. Phys. 2013, 413, 29.

[28] R. D. Mehlenbacher , J. Wang , N. M. Kearns , M. J. Shea , J. T. Flach , T. J. McDonough , M. Y. Wu , M. S. Arnold , M. T. Zanni , J. Phys. Chem. Lett. 2016, 7, 2024. 27182690 10.1021/acs.jpclett.6b00650

[29] M. Gong , T. A. Shastry , Y. Xie , M. Bernardi , D. Jasion , K. A. Luck , T. J. Marks , J. C. Grossman , S. Ren , M. C. Hersam , Nano Lett. 2014, 14, 5308. 25101896 10.1021/nl5027452

[30] Z. Li , H. C. Wong , Z. Huang , H. Zhong , C. H. Tan , W. C. Tsoi , J. S. Kim , J. R. Durrant , J. T. Cabral , Nat. Commun. 2013, 4, 2227. 23892424 10.1038/ncomms3227

[31] Q. Burlingame , X. R. Tong , J. Hankett , M. Slootsky , Z. Chen , S. R. Forrest , Energy Environ. Sci. 2015, 8, 1005.

[32] S. H. K. Paleti , S. Hultmark , N. Ramos , N. Gasparini , A.-H. Emwas , J. Martin , C. Müller , D. Baran , Sol. RRL 2022, 2200436.

[33] Y. He , Y. Li , Phys. Chem. Chem. Phys. 2011, 13, 1970. 21180723 10.1039/c0cp01178a

[34] J. Lee , S. Song , J. Huang , Z. Du , H. Lee , Z. Zhu , S.-J. Ko , T.-Q. Nguyen , J. Y. Kim , K. Cho , G. C. Bazan , ACS Mater. Lett. 2020, 2, 395.

[35] J. Yan , X. Rodriguez-Martinez , D. Pearce , H. Douglas , D. Bili , M. Azzouzi , F. Eisner , A. Virbule , E. Rezasoltani , V. Belova , B. Dorling , S. Few , A. A. Szumska , X. Hou , G. Zhang , H. L. Yip , M. Campoy-Quiles , J. Nelson , Energy Environ. Sci. 2022, 15, 2958. 35923416 10.1039/d2ee00887d PMC9277517

[36] Q. S. Liu , Y. F. Jiang , K. Jin , J. Q. Qin , J. G. Xu , W. T. Li , J. Xiong , J. F. Liu , Z. Xiao , K. Sun , S. F. Yang , X. T. Zhang , L. M. Ding , Sci. Bull. 2020, 65, 272. 10.1016/j.scib.2020.01.001 36659090

[37] L. Zhan , S. Li , X. Xia , Y. Li , X. Lu , L. Zuo , M. Shi , H. Chen , Adv. Mater. 2021, 33, 2007231. 10.1002/adma.202007231 33598972

[38] L. Duan , Y. Zhang , H. Yi , F. Haque , R. Deng , H. Guan , Y. Zou , A. Uddin , Energy Technol. 2019, 8, 1900924.

[39] X. Xu , C. Sun , J. Jing , T. Niu , X. Wu , K. Zhang , F. Huang , Q. Xu , J. Yuan , X. Lu , Y. Zhou , Y. Zou , ACS Appl. Mater. Interfaces 2022, 14, 36582. 35938933 10.1021/acsami.2c07883

[40] T. Yan , W. Song , J. Huang , R. Peng , L. Huang , Z. Ge , Adv. Mater. 2019, 31, 1902210. 10.1002/adma.201902210 31411359

[41] M. Zhang , L. Zhu , G. Zhou , T. Hao , C. Qiu , Z. Zhao , Q. Hu , B. W. Larson , H. Zhu , Z. Ma , Z. Tang , W. Feng , Y. Zhang , T. P. Russell , F. Liu , Nat. Commun. 2021, 12, 309. 33436638 10.1038/s41467-020-20580-8 PMC7803987

[42] G. Yao , Y. S. Ge , X. Y. Xiao , L. F. Zhang , N. Yi , H. Q. Luo , S. S. Yuan , W. H. Zhou , ACS Appl. Energy Mater. 2022, 5, 1193.

[43] Y. Su , L. Zhang , Z. Ding , Y. Zhang , Y. Wu , Y. Duan , Q. Zhang , J. Zhang , Y. Han , Z. Xu , R. Zhang , K. Zhao , S. Liu , Adv. Energy Mater. 2022, 12, 2103940.

[44] X. He , C. C. S. Chan , J. Kim , H. Liu , C. J. Su , U. S. Jeng , H. Su , X. Lu , K. S. Wong , W. C. H. Choy , Small Methods 2022, 6, 2101475. 10.1002/smtd.202101475 35064775

[45] J. Wang , S. R. Peurifoy , M. T. Bender , F. Ng , K.-S. Choi , C. Nuckolls , M. S. Arnold , J. Phys. Chem. C 2019, 123, 21395.

[46] A. Nish , J. Y. Hwang , J. Doig , R. J. Nicholas , Nat. Nanotechnol. 2007, 2, 640. 18654390 10.1038/nnano.2007.290

[47] S. K. Samanta , M. Fritsch , U. Scherf , W. Gomulya , S. Z. Bisri , M. A. Loi , Acc. Chem. Res. 2014, 47, 2446. 25025887 10.1021/ar500141j

[48] A. Graf , Y. Zakharko , S. P. Schiessl , C. Backes , M. Pfohl , B. S. Flavel , J. Zaumseil , Carbon 2016, 105, 593.

[49] J. T. Flach , J. Wang , M. S. Arnold , M. T. Zanni , J. Phys. Chem. Lett. 2020, 11, 6016. 32639162 10.1021/acs.jpclett.0c01555

[50] F. L. Sebastian , N. F. Zorn , S. Settele , S. Lindenthal , F. J. Berger , C. Bendel , H. Li , B. S. Flavel , J. Zaumseil , J. Phys. Chem. Lett. 2022, 3542. 35420437 10.1021/acs.jpclett.2c00758 PMC9059186

[51] L. Wieland , H. Li , C. Rust , J. Chen , B. S. Flavel , Adv. Energy Mater. 2020, 11, 2002880.

[52] Y. Joo , G. J. Brady , M. J. Shea , M. B. Oviedo , C. Kanimozhi , S. K. Schmitt , B. M. Wong , M. S. Arnold , P. Gopalan , ACS Nano 2015, 9, 10203. 26348205 10.1021/acsnano.5b03835

[53] A. T. Mallajosyula , W. Nie , G. Gupta , J. L. Blackburn , S. K. Doorn , A. D. Mohite , ACS Nano 2016, 10, 10808. 27966903 10.1021/acsnano.6b04885

[54] M. Pfohl , K. Glaser , A. Graf , A. Mertens , D. D. Tune , T. Puerckhauer , A. Alam , L. Wei , Y. Chen , J. Zaumseil , A. Colsmann , R. Krupke , B. S. Flavel , Adv. Energy Mater. 2016, 6, 1600890.

[55] T. A. Shastry , M. C. Hersam , Adv. Energy Mater. 2017, 7, 1601205.

[56] L. Lüer , S. Hoseinkhani , D. Polli , J. Crochet , T. Hertel , G. Lanzani , Nat. Phys. 2008, 5, 54.

[57] F. Wang , G. Dukovic , L. E. Brus , T. F. Heinz , Science 2005, 308, 838. 15879212 10.1126/science.1110265

[58] R. Ihly , K. S. Mistry , A. J. Ferguson , T. T. Clikeman , B. W. Larson , O. Reid , O. V. Boltalina , S. H. Strauss , G. Rumbles , J. L. Blackburn , Nat. Chem. 2016, 8, 603. 27219706 10.1038/nchem.2496

[59] X. Liu , X. Li , Y. Zou , H. Liu , L. Wang , J. Fang , C. Yang , J. Mater. Chem. A 2019, 7, 3336.

[60] M. Zheng , B. A. Diner , J. Am. Chem. Soc. 2004, 126, 15490. 15563177 10.1021/ja0457967

[61] L. Lüer , J. Crochet , T. Hertel , G. Cerullo , G. Lanzani , ACS Nano 2010, 4, 4265. 20518568 10.1021/nn100674h

[62] R. D. Mehlenbacher , T. J. McDonough , M. Grechko , M. Y. Wu , M. S. Arnold , M. T. Zanni , Nat. Commun. 2015, 6, 6732. 25865487 10.1038/ncomms7732

[63] A. M. Dowgiallo , K. S. Mistry , J. C. Johnson , J. L. Blackburn , ACS Nano 2014, 8, 8573. 25019648 10.1021/nn503271k

[64] S. L. Guillot , K. S. Mistry , A. D. Avery , J. Richard , A. M. Dowgiallo , P. F. Ndione , J. van de Lagemaat , M. O. Reese , J. L. Blackburn , Nanoscale 2015, 7, 6556. 25790468 10.1039/c5nr00205b

[65] M. Grechko , Y. Ye , R. D. Mehlenbacher , T. J. McDonough , M. Y. Wu , R. M. Jacobberger , M. S. Arnold , M. T. Zanni , ACS Nano 2014, 8, 5383. 24806792 10.1021/nn4041798

[66] J. Yuan , Y. Zhang , L. Zhou , G. Zhang , H.-L. Yip , T.-K. Lau , X. Lu , C. Zhu , H. Peng , P. A. Johnson , M. Leclerc , Y. Cao , J. Ulanski , Y. Li , Y. Zou , Joule 2019, 3, 1140.

[67] Z. Zhang , L. Li , C. Xu , P. Jin , M. Huang , Y. Li , H. Wang , Y. Yi , C. Zhang , Y. Yang , W. Xu , Y. Lin , Cell Rep. Phys. Sci. 2022, 3, 100895.

[68] Y. Cai , Q. Li , G. Lu , H. S. Ryu , Y. Li , H. Jin , Z. Chen , Z. Tang , G. Lu , X. Hao , H. Y. Woo , C. Zhang , Y. Sun , Nat. Commun. 2022, 13, 2369. 35501300 10.1038/s41467-022-29803-6 PMC9061803

[69] Y. Firdaus , V. M. Le Corre , S. Karuthedath , W. Liu , A. Markina , W. Huang , S. Chattopadhyay , M. M. Nahid , M. I. Nugraha , Y. Lin , A. Seitkhan , A. Basu , W. Zhang , I. McCulloch , H. Ade , J. Labram , F. Laquai , D. Andrienko , L. J. A. Koster , T. D. Anthopoulos , Nat. Commun. 2020, 11, 5220. 33060574 10.1038/s41467-020-19029-9 PMC7562871

[70] O. V. Mikhnenko , P. W. M. Blom , T.-Q. Nguyen , Energy Environ. Sci. 2015, 8, 1867.

[71] M. T. Sajjad , A. Ruseckas , I. D. W. Samuel , Matter 2020, 3, 341.

[72] X. Li , R. Zhu , Z. He , X. Du , H. Lin , C. Zheng , G. Yang , Z. Chen , S. Tao , ACS Appl. Mater. Interfaces 2022, 14, 25842. 35635178 10.1021/acsami.2c04997

[73] A. Yi , S. Chae , H. Yoon , H. J. Kim , ACS Appl. Mater. Interfaces 2021, 13, 60288. 34889097 10.1021/acsami.1c18952

[74] N. Tokmoldin , S. M. Hosseini , M. Raoufi , L. Phuong , O. J. Sandberg , H. L. Guan , Y. P. Zou , D. Neher , S. Shoaee , J. Mater. Chem. A 2020, 8, 7854.

