# Supplementary material for: Ternary PM6:Y6 Solar Cells with Single‐Walled Carbon Nanotubes
Source: Small Sci. 2022 Dec 25;3(2):2200079. doi: 10.1002/smsc.202200079 (PMC11935985; doi:10.1002/smsc.202200079)
Supplement: Supplementary file 1 — Supplementary Material [file SMSC-3-2200079-s001.pdf]

Supporting Information

Ternary PM6:Y6 Solar Cells with Single Wall Carbon Nanotubes

Laura Wieland, Han Li, Xuning Zhang, Jianhui Chen & Benjamin S. Flavel**

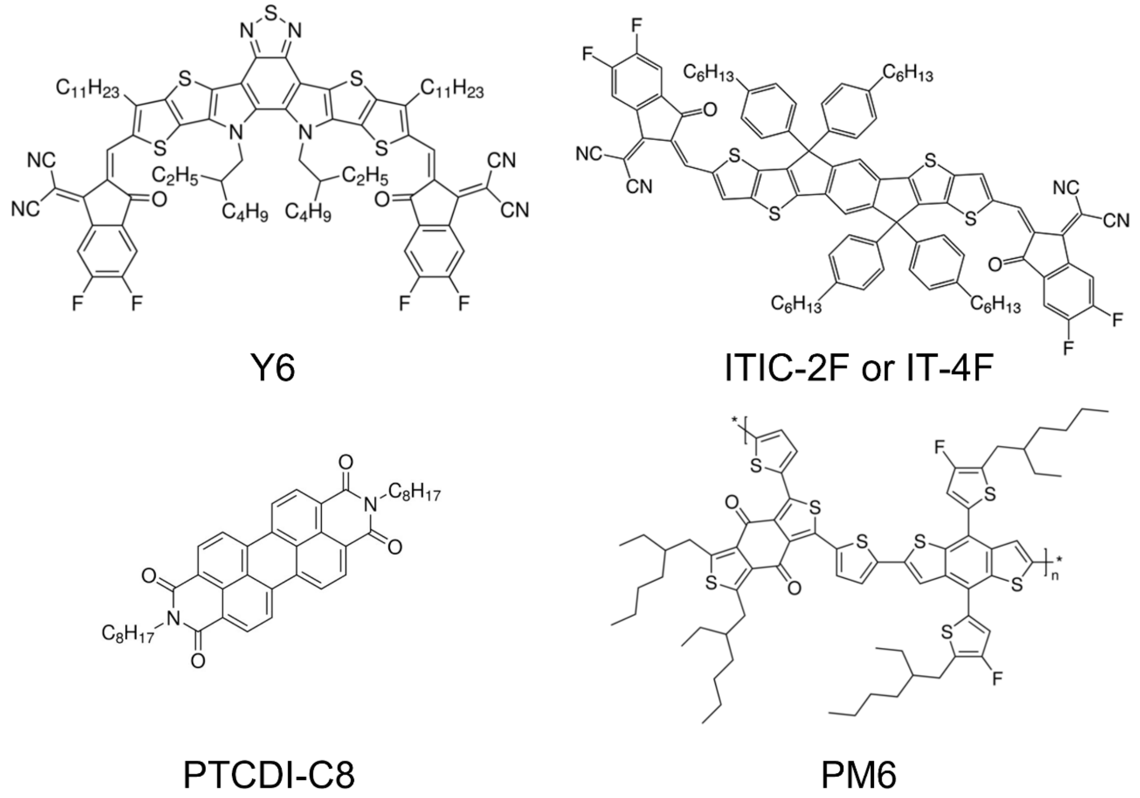

Figure S1. Chemical structures of the non-fullerene acceptors ITIC-2F (or IT-4F), Y6 and PTCDI-C8 and the donor PM6.

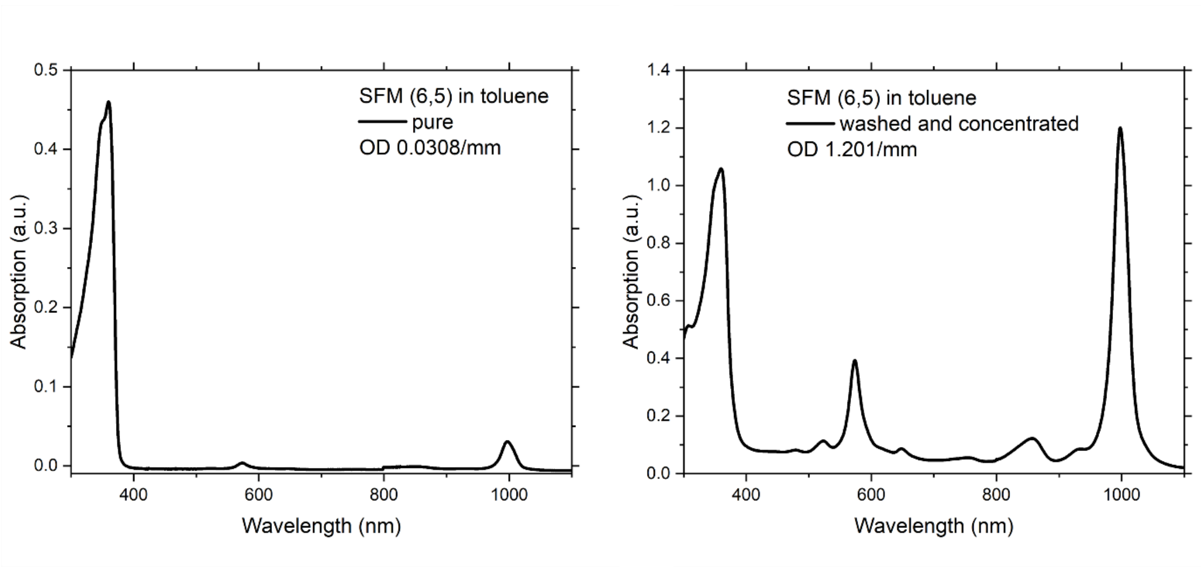

Figure S2. Absorption spectra of (a) (6,5) SWCNTs in toluene directly after shear-force mixing (SFM) and (b) after an additional concentration and washing step to remove most of the wrapping polymer PFO-BPy. Removal of excess PFO-BPy is evidenced by a reduction in the peak at 360 nm.

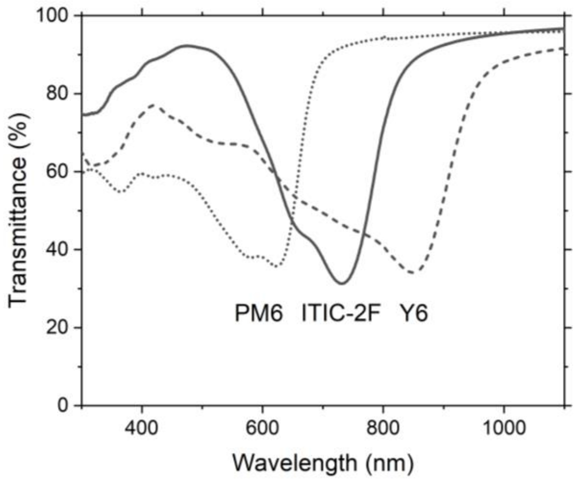

Figure S3. Film transmittance spectra of PM6, ITIC-2F and Y6 on glass.

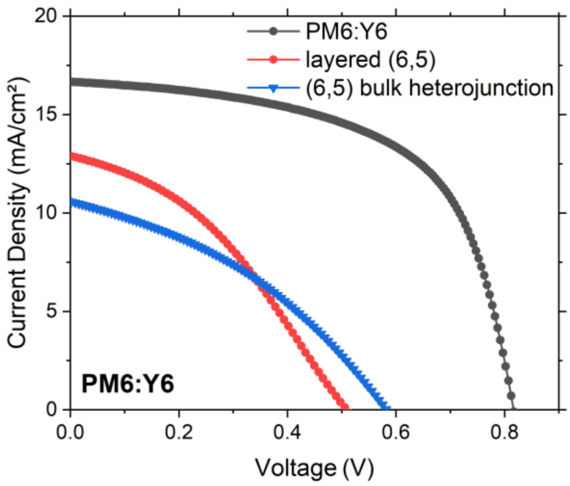

Figure S4. J-V measurements of the reference PM6:Y6 heterojunction in comparison to devices including (6,5) SWCNTs either as a layer or in bulk.

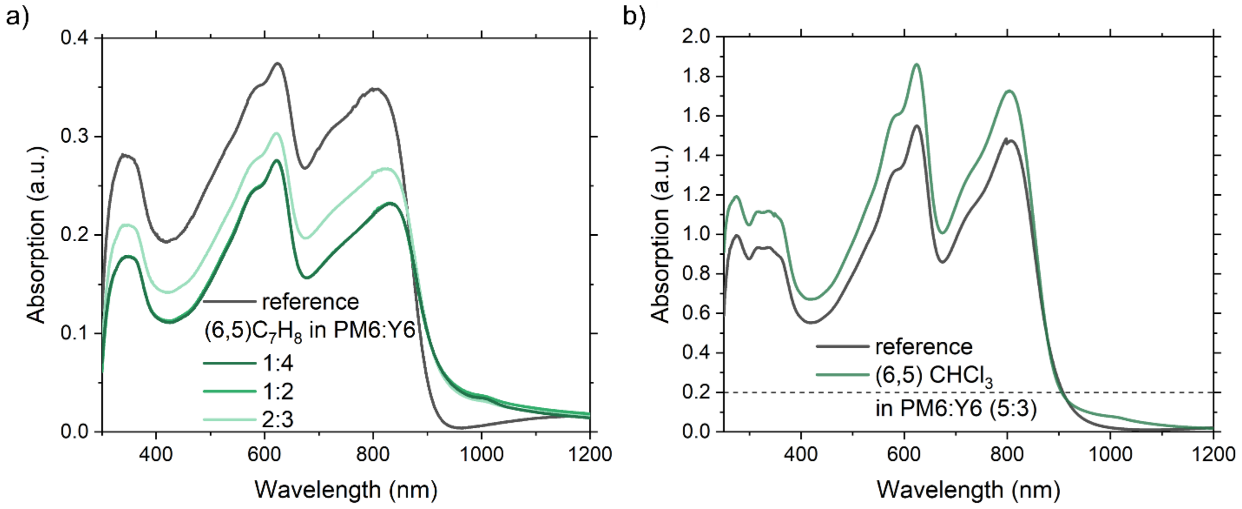

Figure S5. Absorption spectra of films of (a) (6,5) SWCNTs in toluene (C_7H_8) added directly to PM6:Y6 in chloroform and (b) with (6,5) SWCNTs in chloroform and used to disperse PM6:Y6 in a ratio of 5:3. The dashed line at 0.2 OD highlights the maximum S_{11} absorptivity of SFM (6,5) films as shown in Figure 3 (c).

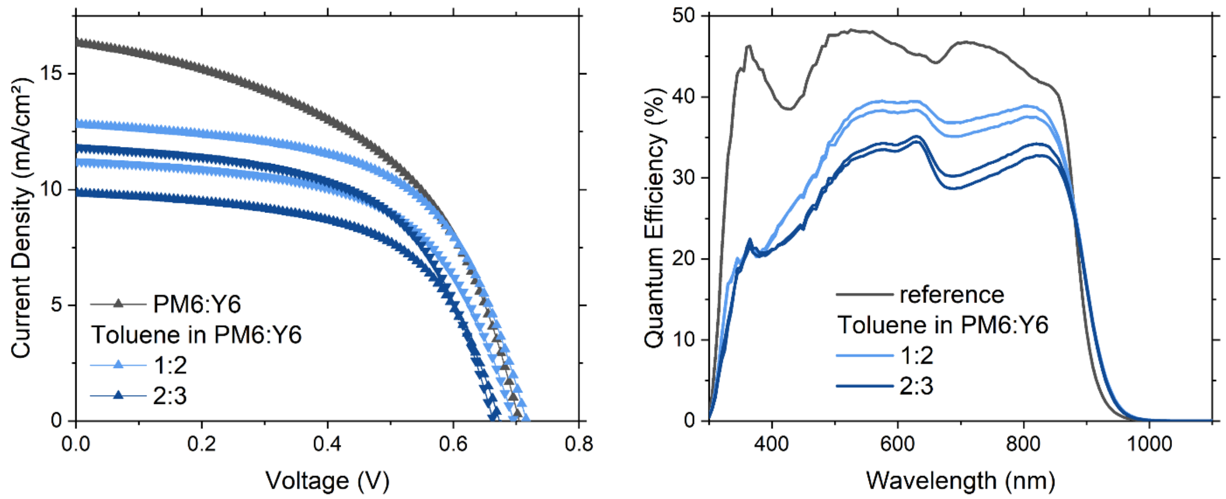

Figure S6: J-V measurements of the reference PM6:Y6 heterojunction in comparison to bulk heterojunctions with additional toluene (but no carbon nanotubes) in the ratios 1:2 and 2:3.

Table S1: Solar cell performance parameters for bulk heterojunction ITO/PEDOT:PSS/C_7H_8:PM6:Y6/BCP/Ag devices with varying toluene content.

**Layer stack**<br>ITO/PEDOT:PSS/…/ BCP/Ag	**J_{SC}**<br>**[mA/cm²]**	**V_{OC}**<br>**[mV]**	**FF**<br>**[%]**	**Efficiency**<br>**[%]**
PM6:Y6	16.30	703.9	49.03	5.62
C_7H_8 in PM6:Y6 (1:2)	12.80	715.6	57.89	5.30
	11.21	695.9	57.75	4.51
C_7H_8 in PM6:Y6 (2:3)	9.84	672.8	58.34	3.86
	11.82	662.3	57.34	4.49

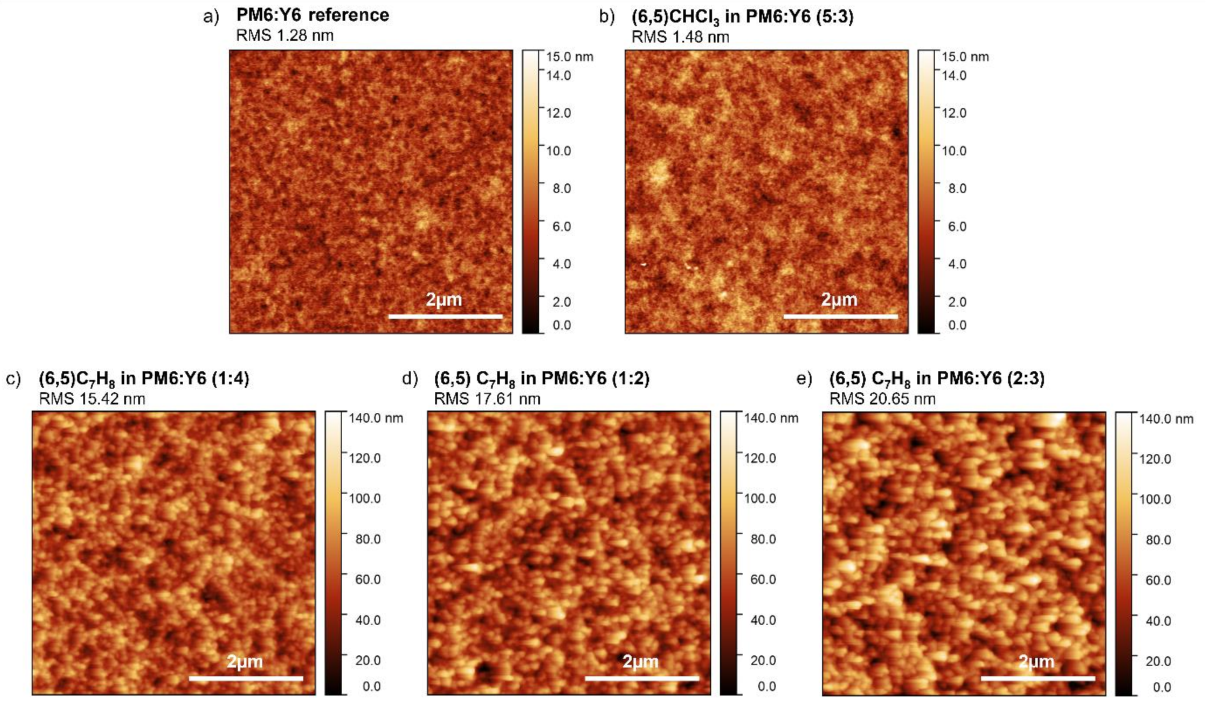

Figure S7. AFM images of PM6:Y6 spin case onto a glass substrate and with the addition of (6,5) SWCNTs either in toluene (C_7H_8) or chloroform ($CHCl_3$).
